# Mini Review: Impedance Measurement in Neuroscience and Its Prospective Application in the Field of Surgical Neurooncology

**DOI:** 10.3389/fneur.2021.825012

**Published:** 2022-01-17

**Authors:** Tammam Abboud, Dorothee Mielke, Veit Rohde

**Affiliations:** Department of Neurosurgery, University Medical Center Göttingen, Göttingen, Germany

**Keywords:** impedance, resistivity, brain tumors, glioma, surgery

## Abstract

Impedance measurement of human tissue can be performed either *in vivo* or *ex vivo*. The majority of the *in-vivo* approaches are non-invasive, and few are invasive. To date, there is no gold standard for impedance measurement of intracranial tissue. In addition, most of the techniques addressing this topic are still experimental and have not found their way into clinical practice. This review covers available impedance measurement approaches in the neuroscience in general and specifically addresses recent advances made in the application of impedance measurement in the field of surgical neurooncology. It will provide an understandable picture on impedance measurement and give an overview of limitations that currently hinders clinical application and require future technical and conceptual solutions.

## Introduction

The electrical conductivity of a biological tissue is determined by its molecular composition, cellular structure, amounts of intra- and extra-cellular fluids, concentration and mobility of ions in those fluids, temperature and other factors (1–3). Impedance, from an electrical point of view, is the obstruction to the flow of an alternating current and is dependent on the frequency of the applied current (4, 5). Bioimpedance is composed of resistance, which is caused by water content and reactance, which is caused by the capacitance of the cell membrane (4, 6). The estimation of tissue impedance has been a matter of research for decades. It has sparked interest in many medical fields (7). Especially in the domain of oncology, impedance measurement has been successfully applied for diagnostic purposes to distinguish cancerous from benign tissue, e.g., in breast and prostate cancers (8, 9). This application is based on the assumption that cancerous tissue exhibits changes in the ions concentration and consequently in the water content, leading to a change in electrical conductivity (7). In the neurosciences, there has been different approaches to study electrical properties of intracranial tissues. These approaches had to deal with many obstacles, which are mainly related to physiological barriers surrounding the brain as well as anatomical and functional considerations. In this mini review, we will give a summary of the available approaches on investigating electrical properties of human brain. In addition, we will address recent advances in studying tissue impedance of the brain in the field of neurooncology.

## Techniques of Measuring Bioimpedance of Intracranial Tissues

Impedance measurement *per se* is not technically demanding. However, this changes when it comes to bioimpedance as several limitations have to be considered depending on the setting in which the measurement is performed. We therefore divided approaches to measure impedance into non-invasive ones, that are performed non-invasively and without exposing intracranial tissues, and intraoperative approaches that applies invasive measurement or require exposing intracranial tissues.

### Non-invasive Approaches

Studying electrical impedance of human brain tissues has been mainly based on non-invasive methods. In general, a current is injected through the scalp and current distribution within the skull is interpreted through recordings on the skull with EEG-electrodes. Electrical impedance tomography (EIT) is an *in-vivo* approach that relies on this principle and enables the internal impedance of an object to be imaged non-invasively (10). Although it has not found a way into clinical practice yet, EIT might enable imaging of brain function and pathology (11). Its experimental applications currently include localization of epileptic foci (12, 13) and monitoring cerebral edema, ischemia and intracranial hemorrhage (14–17). Notably in combination with stereoelectroencephalography (SEEG) (18) in the presurgical work-up for epilepsy, EIT might add new information or be even superior to the available imaging modalities in defining epileptic foci. Apart from epileptology, the question whether EIT would add a value to existing imaging tools such as CT and MRI scans, particularly in the neurooncology, is justified, given that EIT exhibits significant limitations. On the one hand, scalp and skull are known to diminish the amplitude of the signal (19) and on the other hand the amount of current passing through the brain is probably too small compared to the current shunted by the scalp and therefore the sensitivity to resistivity differences within the brain might be insufficient (13). Romsauerova et al. applied multi-frequency EIT in seven human subjects with brain tumors, arteriovenous malformations or chronic stroke and observed no reproducible changes between pathologies (20). They explained their negative results by the fact that the variability induced by the method itself might be higher than the presumable differences between pathologies.

A further non-invasive approach that was proposed to deal with technical difficulties of EIT and produce images with a spatial resolution is magnetic resonance electrical impedance tomography (MREIT) (3). It is an imaging technique that reconstructs the conductivity distribution inside the subject using magnetic flux density or current density measurements acquired by a magnetic resonance imaging system (14, 15). Algorithms are currently being developed to optimize the use of acquired images to reconstruct electric field and current density distributions (21). In a similar way, MR- electrical properties tomography (EPT) non-invasively images the conductivity and permittivity maps *in vivo* from the radiofrequency field signals obtained with MRI. Unlike the MREIT, EPT does not induce additional external energy other than the inherent radiofrequency fields. However, due to the large number of EPT approaches with a large variety in requirements, assumptions and complexity, and since the EPT field is relatively new, most methods are not at the stage of clinical use yet (22).

### Intraoperative Approaches and Prospective Role of Impedance Measurement in Surgical Neurooncology

Therapeutic approaches to treat brain tumors have mainly addressed their biological properties. The electrical properties of brain tumors are largely unknown. The reason for this is that to date no suitable technique has been developed for performing an *in-vivo* measurement of the electrical resistivity of brain and tumor tissue, nor are normative values available for resistivity of various brain tissues, including white matter. Although *ex-vivo* measurement of electrical resistivity is a feasible technique, its results, however, do not reflect necessarily the real *in-vivo* resistivity values of intracranial tissues. This can be attributed to the fact that cell death starts directly after tissue excision and leads to an increase in tissue resistivity (23). In addition, electrical resistivity is known to correlate with blood perfusion into tissue (24), which stops as soon as the tissues are removed. Approaches to directly measure tissue resistivity during brain surgery are scarce. The first one was reported by Latikka et al., who performed resistivity measurement using a monopolar needle electrode in nine patients. Mean resistivity values were 3.5 Ω^*^m for gray matter and 3.9 Ω^*^m for white matter. Values for tumor tissues ranged from 2.3 to 9.7 Ω^*^m (25).

The second approach was introduced by Koessler et al. who performed measurements of human brain tissue resistivity using intracerebral multicontact electrodes designed for SEEG in fifteen epileptic patients. The electrodes were placed in a stereotactic procedure. They found mean resistivity values of 3.8 Ω^*^m for gray matter, 5.2 Ω^*^m for white matter and 3.5 Ω^*^m for epileptogenic zones (26). A new method was proposed to use EIT in combination with SEEG. It was applied in 15 cases and the results suggested that adding EIT to SEEG measurements might improve the diagnostic yield in epilepsy (18).

**Figure 1:**
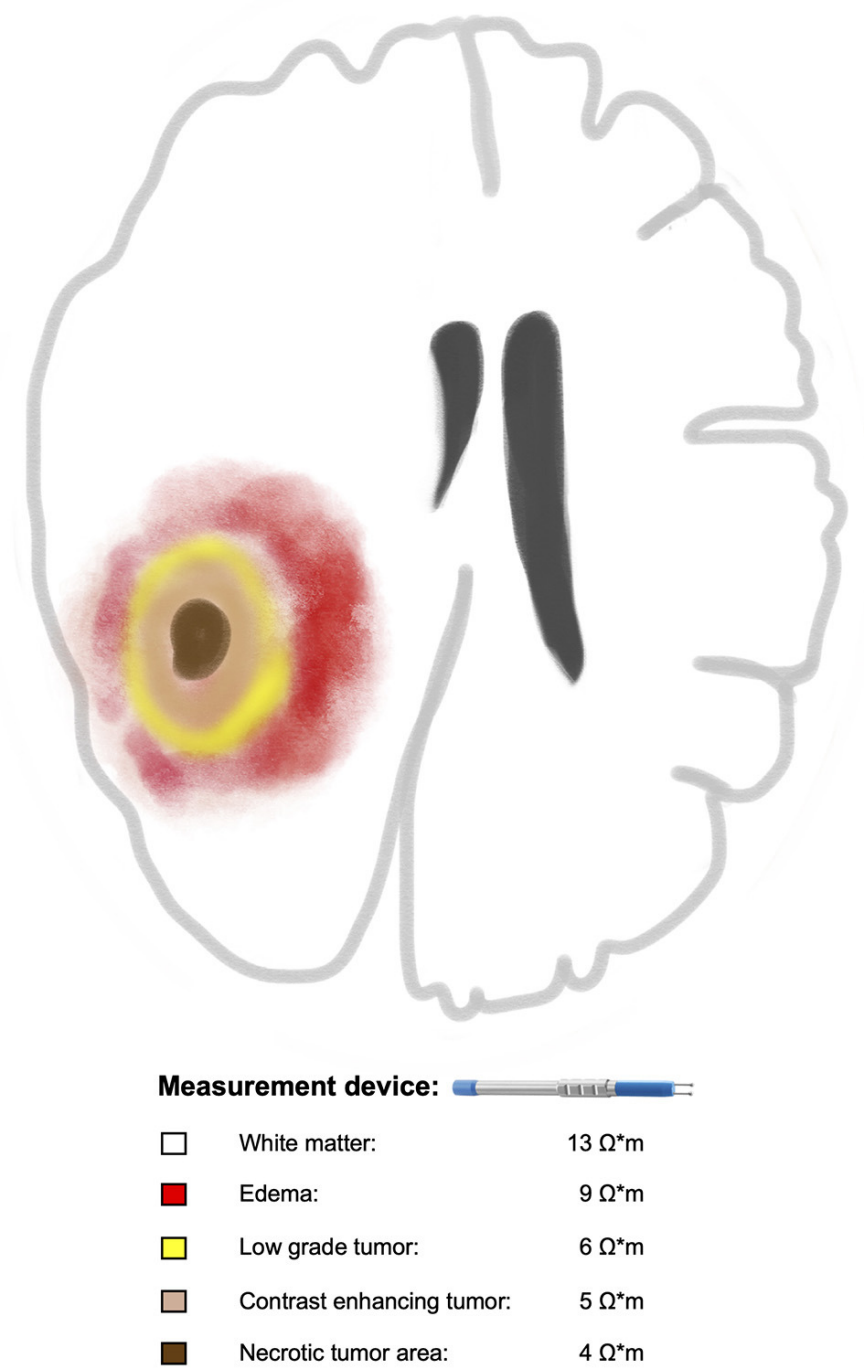
Illustration of a glioma with the assumed correlation between increasing tumor malignancy and decreasing electrical resistivities of the tumor tissue in comparison to surrounding white matter. Braun color represents necrotic tumor area with the lowest resistivity value (4 Ω^*^m) followed by contrast enhancing tumor area in beige (5 Ω^*^m), low grade part in yellow (6 Ω^*^m), edema in red (9 Ω^*^m) and white matter outside the edema in white (13 Ω^*^m). For reference, please see the text.

The third approach was recently presented by our group in a prospective cohort of ninety-two patients who underwent surgery for brain tumors (27). The aim of the study was to investigate the feasibility of *in vivo* measurement of electrical impedance during tumor resection. Moreover, we intended to investigate potential differences between tumor tissue and white matter as well as between different tumor subtypes. For this purpose, we used a bipolar probe that is approved for brain stimulation to measure the electrical impedance and the tips of the bipolar probe were half embedded in the exposed tissue during surgery. The measurement was performed in the white matter within and outside peritumoral edema as well as in non-enhancing, enhancing and necrotic tumor areas. The position of the probe within the studied tissue was confirmed using MRI-based intraoperative neuronavigation that was installed before skin incision. The probe was calibrated *ex-vivo* and with a simulation program in order to calculate the electrical resistivity. White matter outside peritumoral edema had higher resistivity values (13.3 ± 1.7 Ω^*^m) than within peritumoral edema (8.5 ± 1.6 Ω^*^m), and both had higher values than brain tumors including low grade gliomas (6.4 ± 1.3 Ω^*^m) and enhancing areas in glioblastomas (WHO IV:5 ± 1 Ω^*^m) (27). In addition, no overlap was found between resistivity values of brain and tumor tissues stemming from the same patient. Resistivity values of white matter were on average 158% higher than the highest tumor values. Resistivity of edema was on average 85% higher than the highest tumor values. Resistivity of white matter was on average 60% higher than resistivity of edema. Thus, our results suggested that tumor tissue, depending on the degree of malignancy, can differ in its electrical resistivity from surrounding healthy tissue (Figure 1). These finding, if further verified, might create the basis for a resistivity-guided tumor resection.

## Discussion

Impedance measurement currently seems to be a promising tool in the field of epilepsy surgery. Non-invasively, it can add to the armamentarium of the currently present tools (e.g., EEG, subdural grids, and SEEG) to identify epileptogenic area (18, 28). Invasively, it harbors potential to help the surgeon identify epileptogenic zones during resection (26). Large and perhaps multi-center clinical trials are needed to move the application of impedance measurement in epilepsy surgery into routine clinical practice.

There is so far no published data that confirms a potential role of EIT in diagnostic or treatment of brain tumors. Other impedance-based imaging such as MREIT and EPT have not reached a stage of being a useful supplement to the available imaging modalities. Moreover, it also seems that technical breakthroughs are needed to make these techniques of a clinical merit (22, 29).

Through recent reports, a new possibility to understand electrical properties of brain tissue and tumors is emerging. Intraoperative impedance measurement at exposed brain und tumor tissues seems to be feasible. Its results can fundamentally change the role of impedance from a purely diagnostic tool toward one that offer direct assistance during tumor resection. One has to mention that our intraoperative approach, as well as previously described intraoperative approaches were limited by the fact that impedance measurement was not performed at a wide range of frequencies, which would be necessary to involve extra- and intracellular compartments in the impedance measurement (30). In addition, the measurements were undertaken at certain spots within the brain, which limits the ability to generalize the obtained resistivity values. Moreover, neither of those approaches considered the anisotropy of the white matter that is supposed to affect impedance measurement, even though, the range within which the anisotropy would affect results of electrical impedance measurement remains unknown. A further hurdle is that calibration and/or calculation the geometry of the used probe to estimate electrical resistivity was always necessary, because the ideal technique to measure electrical resistivity with the classical 4-point probes method is currently not feasible in the setting of brain surgery. Therefore, technical and conceptual solutions will be needed to overcome these limitations and allow for future application of the emerging knowledge about electrical resistivity in the field of neurooncology.

In summary, impedance measurement still has a long way to go, before it can be implemented in the clinical practice of neurooncology. A gold standard to measure tissue impedance within the brain has not been established yet. In addition to producing imaging, impedance measurement has the potential to play a role in identifying tumor tissue during surgical resection of brain tumors. Therefore, research efforts should pay more attention to this important aspect.

## Author Contributions

TA wrote the manuscript. DM and VR reviewed and edited the manuscript. All authors contributed to the article and approved the submitted version.

## Funding

Funding was received from the research program, University Medical Center, University of Goettingen.

## Conflict of Interest

The authors declare that the research was conducted in the absence of any commercial or financial relationships that could be construed as a potential conflict of interest.

## Publisher's Note

All claims expressed in this article are solely those of the authors and do not necessarily represent those of their affiliated organizations, or those of the publisher, the editors and the reviewers. Any product that may be evaluated in this article, or claim that may be made by its manufacturer, is not guaranteed or endorsed by the publisher.
